# Observations on the expression of human papillomavirus major capsid protein in HeLa cells

**DOI:** 10.1186/s12935-015-0206-0

**Published:** 2015-05-23

**Authors:** Chang-Yi Xiao, Bing-Bing Fu, Zhi-Ying Li, Gohar Mushtaq, Mohammad Amjad Kamal, Jia-Hua Li, Gui-Cheng Tang, Shuo-Shuang Xiao

**Affiliations:** Tumor Research Institute, China Three Gorges University, Yichang, 443002 Hubei Province People’s Republic of China; Department of Gynaecology and Obstetrics, The Second Clinical Medical College of China Three Gorges University, Yichang, 443000 Hubei Province People’s Republic of China; Department of Biochemistry, College of Science, King Abdulaziz University, Jeddah, 21589 Saudi Arabia; King Fahd Medical Research Center, King Abdulaziz University, Jeddah, 21589 Saudi Arabia; Enzymoic, 7 Peterlee Place, Hebersham, NSW 2770 Australia

**Keywords:** HeLa cells, Cervical cancer, Human papillomavirus, Major capsid protein, Expression

## Abstract

**Background:**

The goal of this study was to identify the nature of the inclusion bodies that have been found in HeLa cells (cervical cancer immortal cell line) by electron microscope and to determine whether the major capsid protein (L1) of human papillomavirus (HPV) can be expressed in HPV-positive uterine cervix cancer cells.

**Methods:**

HPV L1 protein expression in HeLa cells was detected with anti-HPV L1 multivalent mice monoclonal antibody and rabbit polyclonal anti-HPV L1 antibody by ELISA, light microscope immunohistochemistry, electron microscope immunocytochemistry and Western blotting assays. Reverse transcriptional PCR (RT-PCR) was performed to detect the transcription of L1 mRNA in HeLa cells. The immortalized human keratinocyte HeCat was used as the negative control.

**Results:**

HPV L1 proteins reacted positively in the lysate of HeLa cells by ELISA assays. HRP labeled light microscope immunohistochemistry assay showed that there was a strong HPV L1 positive reaction in HeLa cells. Under the electron microscope, irregular shaped inclusion bodies, assembled by many small and uniform granules, had been observed in the cytoplasm of some HeLa cells. These granules could be labeled by the colloidal gold carried by HPV L1 antibody. The Western blotting assay showed that there was a L1 reaction strap at 80–85 kDa in the HeLa cell lysates, hence demonstrating the existence of HPV18 L1 in HeLa cells. RT-PCR assay showed that the L1 mRNA was transcribed in HeLa cells.

**Conclusions:**

The inclusion bodies found in the cytoplasm of HeLa cells are composed of HPV18 L1 protein. Since HeLa cell line is a type of cervical cancer cells, this implies that HeLa cells have the ability to express HPV L1 proteins.

## Background

Human papillomavirus major capsid protein (HPV L1) is the main protein constituent of the HPV particle coat. The HPV particle coat is highly immunogenic and it can effectively stimulate the humoral immunity and cellular immunity of human body, thus preventing the body from being re-infected by the same HPV [1–4]. This is the main reason why the L1 protein is used as HPV prophylactic vaccine [5,6]. However, so far no noticeable progress has been made in the use of HPV L1 protein as therapeutic vaccine to treat tumors caused by HPV or using it as the target protein for biotherapy of tumors caused by HPV due to the lack of expression of HPV L1 in tumor cells. Most researchers think that HPV does not cause any L1 protein expression in tumors [7,8]. The L1 expression is dependent on cell differentiation [9–13] but owning to the dedifferentiation of tumor cells, the expression program of the L1 gene cannot be activated even if tumor cells have intact episomal HPV genomes. Hence, there is no L1 protein expression [9–13]. Furthermore, some genes in the virus genome are lost and HPV genes are rearranged when HPV genome is integrated into the nucleotide sequence of the host genome. After the integration, although the L1 gene is intact in the integrated HPV genome, the startup sequence needed for L1 normal transcription undergoes rearrangement from upstream to downstream of L1 gene. Consequently, the transcription of the L1 gene cannot start, thus there is no L1 expression [11–16]. Based on the above-mentioned reasons, it is widely believed by researchers in the field of HPV-associated tumors that there is no L1 protein expression in HPV induced tumors [9–13,16]. But the question arises as to whether the above viewpoint really true? The answer to this question may be “no” based on our observation of the HeLa cells.

The HeLa cell line was derived from a case of adenocarcinoma of the uterine cervix in 1952 and it was established to be the first human cervical epithelium carcinoma cell line with a long term cell culture [17]. The existence of HPV18 DNA in HeLa cell line was confirmed after its identification in a case of cervical cancer viable tissue in 1984. There are two HPV18 DNA genomes in HeLa cell. The length of these two HPV18 DNA genomes is 7.8 kb and 6.9 kb, respectively, after Ecor RI analysis, and there is 900 bp deficiency in the latter [18]. As a representative HPV18 positive human cervix cancer cell line, the HeLa cell is used widely in all kinds of cervix cancer researches and it plays an important role in the research of cervix cancer cell biology and the diagnosis and treatment of cervix cancer. Is there HPV L1 expression in HeLa cell? To the best of our knowledge, no previously reported study on HeLa cells has ever reported HPV L1 expression in HeLa cells [14,17–20]. However, we are reporting in our present research the presence of inclusion bodies of different amount, sizes and shapes in the HeLa cell cytoplasm as seen under the electron microscope. This goal of our research is to identify whether this kind of material is the L1 protein expressed by HPV18 genome.

## Results

### Results of HPV L1 assay by ELISA

Extracted from HeLa cells, the protein was detected using HPV L1 mice antibody to show HPV L1 protein expression. The HaCat cells served as negative control. The results revealed that the OD value of HeLa cells was 0.9056 ± 0.2650 while the OD value of HaCat cells was 0.1694 ± 0.0615, hence showing a significant difference (P < 0.01).

### Results of RT-PCR

**Fig. 1:**
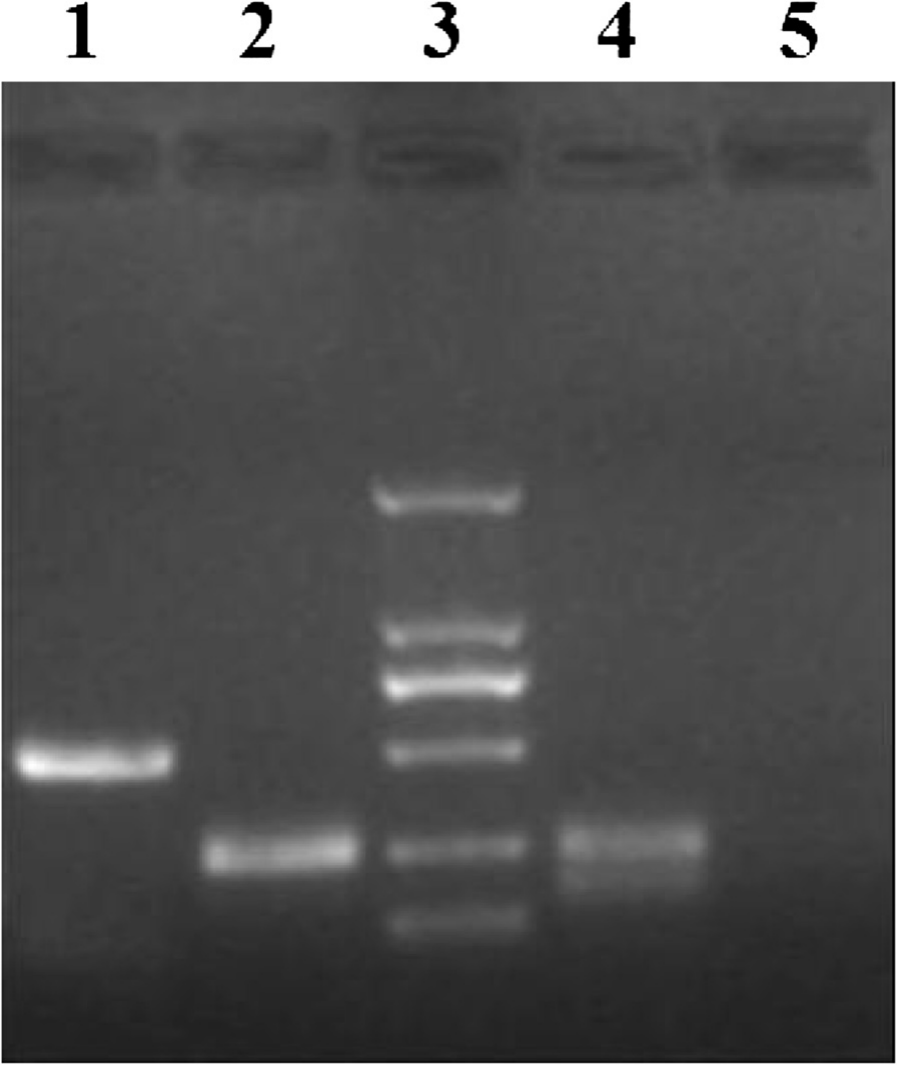
Electropherogram of RT-PCR products. After reverse transcription of mRNA extracted from HeLa cells and HaCat cells, the mRNA were amplified by MY09/11 primer and the products were electrophoresed in sepharose gel. Lane 3 is DNA Marker, from top to bottom are 2000, 1000, 750, 500, 250 and 100 bp. Lane 1 is the amplification products lane of cDNA in HeLa cells. The HPV L1 transcription product strip at 450 bp shows that there is L1 genetic expression in HeLa cells. Lane 2 is the GAPDH internal control lane in HeLa cells and its strip is present at 258 bp. Lane 4 is the GAPDH internal control lane in HaCat cells and its strip is present at 258 bp. Lane 5 is the MY09/11 universal primer amplification compared lane in HaCat cells and there is no amplified strip in this lane

The cultured HeLa cells synthetized cDNA by reverse transcription and amplification reaction. GAPDH internal control products presented a strap at 258 bp when PCR products were in agarose gel electrophoresis, which shows that mRNA extraction and the subsequent operations were performed well. The products produced by synthetized cDNA amplified by MY09/11 universal primer presented a strap at 450 bp after electrophoresis. This strap is produced by the amplification products after HPV L1 universal primer amplification. It indicates the presence of L1 genetic transcription products which come from L1 mRNA produced in the HeLa cells (Fig. 1).

### L1 protein expression assay by light microscope immunocytochemistry

**Fig. 2:**
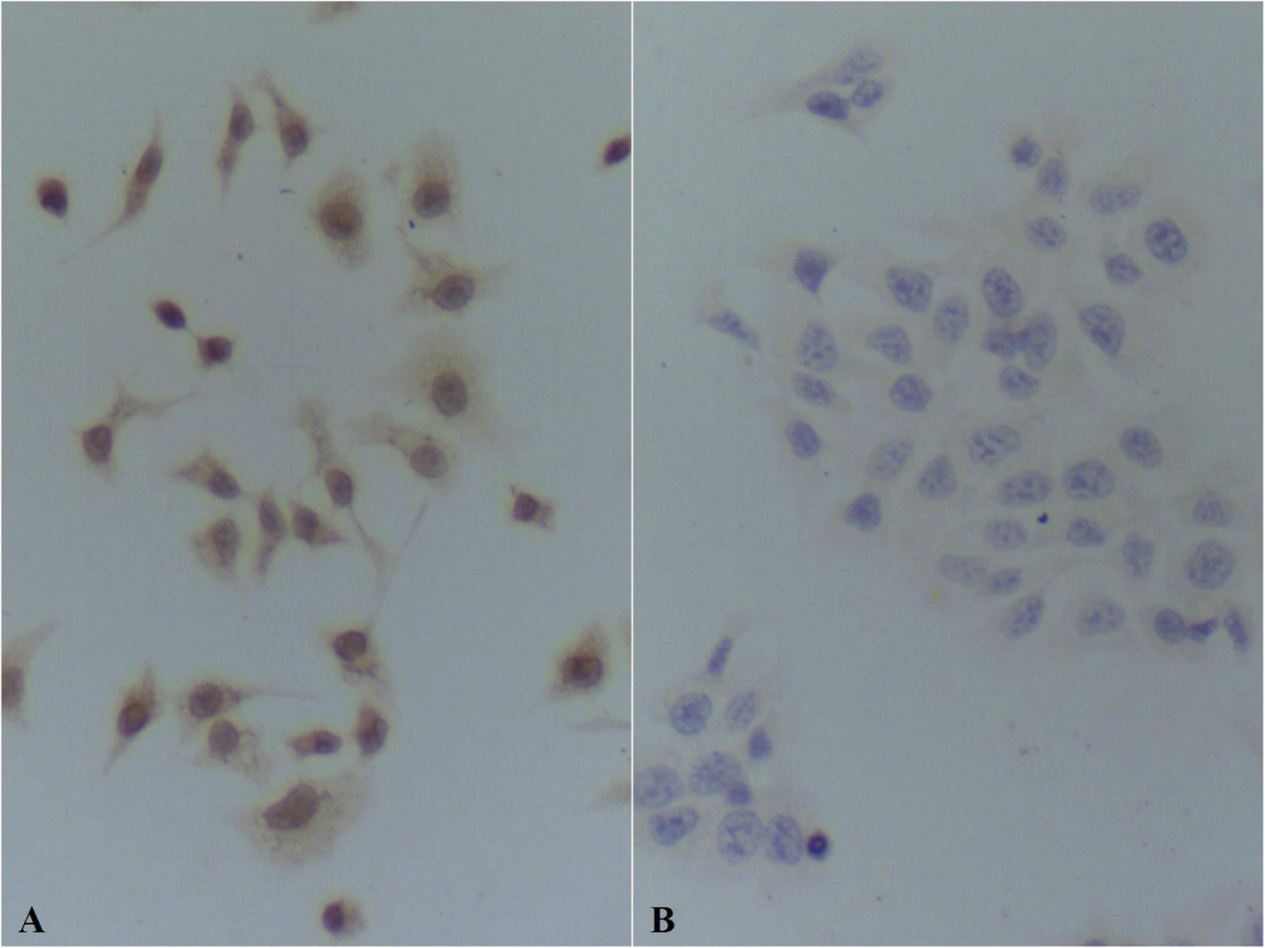
HPV L1 immunocytochemistry assay. **a** HeLa cells. There were positive reactions which were yellow in cytoplasm and nucleus in HeLa cells detected using anti-HPV L1 monoclonal antibody and the stain in cytoplasm is darker. The reaction shows the existence of HPV L1 in HeLa cells. **b** HaCat cells. No positive reaction was detected in HaCat cells using anti-HPV L1 monoclonal antibody

Results of the immunocytochemistry assay for cultured HeLa cells using mice anti-HPV L1 multivalence monoclonal antibody show that HeLa cells in positive reaction which manifests as yellow color all over the whole HeLa cell. The reaction of cytoplasm is stronger and it is of darker stain (Fig. 2a). This result demonstrates the existence of HPV L1 protein in HeLa cells. There is no positive stain reaction in HaCat cells used as negative control after the same assay, which is in sharp contrast to the positive reaction in HeLa cells (Fig. 2b).

### HPV L1 protein expression assay by electron microscope immunocytochemistry

**Fig. 3:**
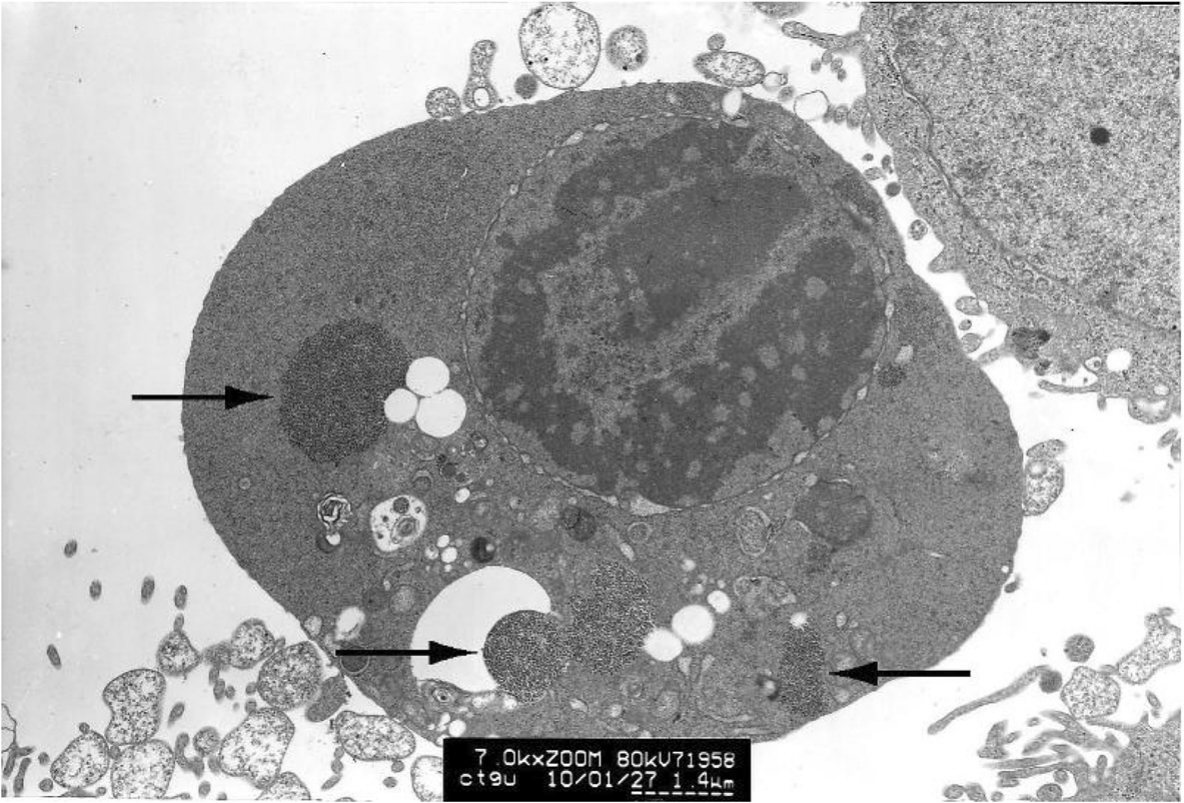
HeLa cell electron micrograph. In the graph, there is a HeLa cell in apoptosis. Many clumped inclusion bodies can be seen in the HeLa cell cytoplasm (indicated by the arrow). These clumped bodies appear in different sizes and shapes. No inclusion bodies are seen in the cell nucleus

**Fig. 4:**
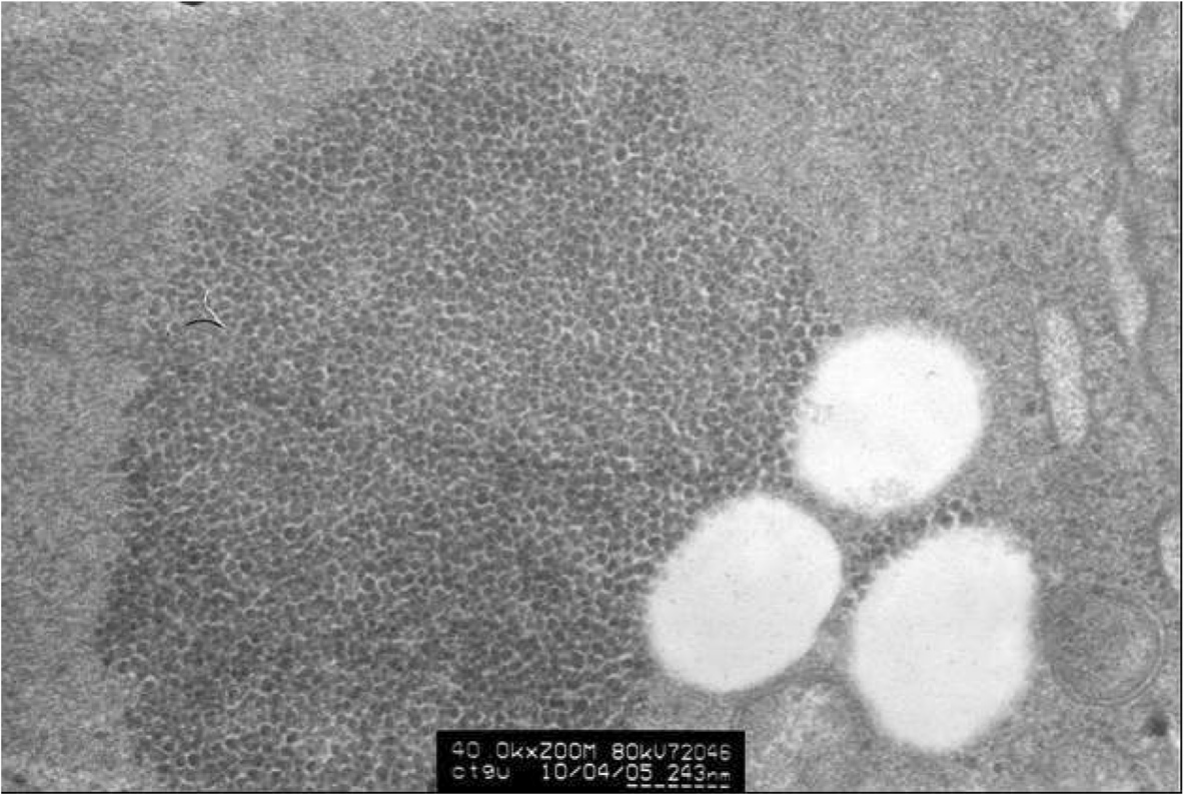
The graph of partial enlargement of the HeLa cell electron micrograph in Fig. 3. Two of the inclusion body structures are highly magnified. At higher magnification, these inclusion bodies can be seen as dense aggregations of plenty of tiny granules with uniform diameter

**Fig. 5:**
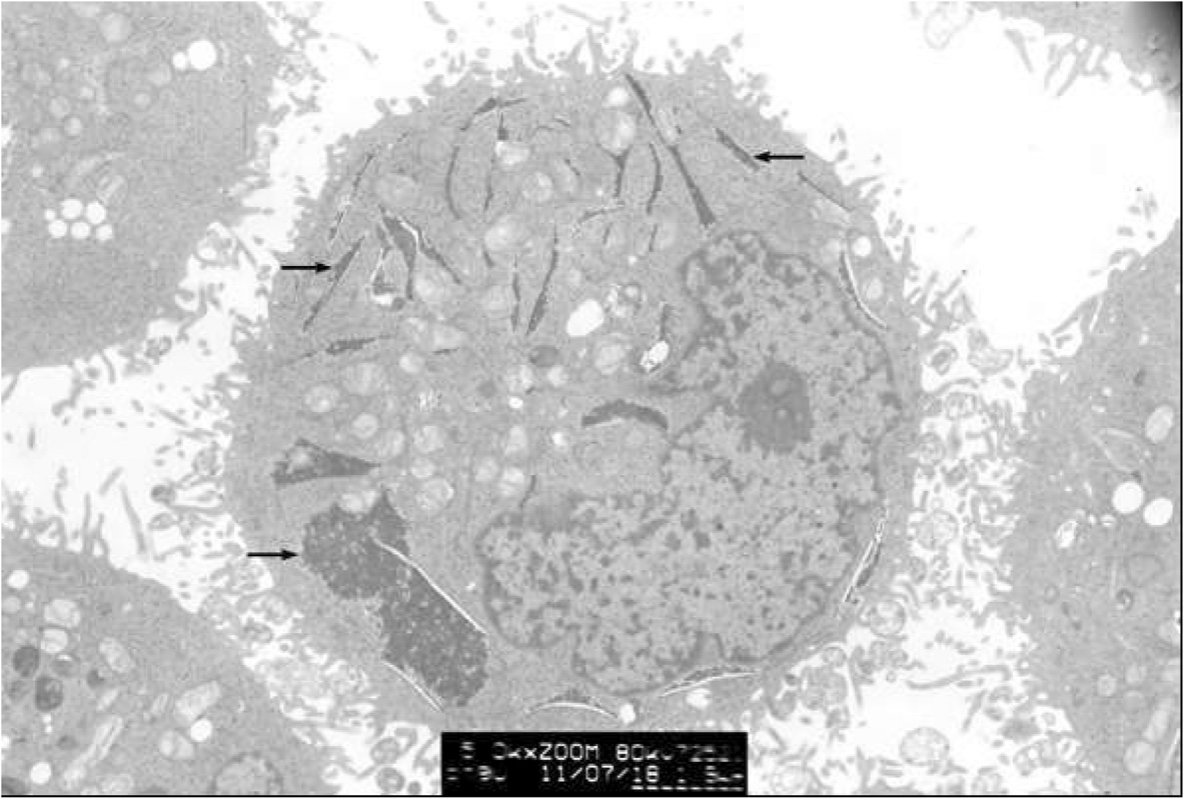
HeLa cell electron micrograph. There is an active HeLa cell. Many clumped inclusion bodies can be seen in the cytoplasm of the HeLa cells (indicated by the arrow). These clumped bodies appear in different sizes and shapes. No inclusion bodies are seen in the cell nucleus

**Fig. 6:**
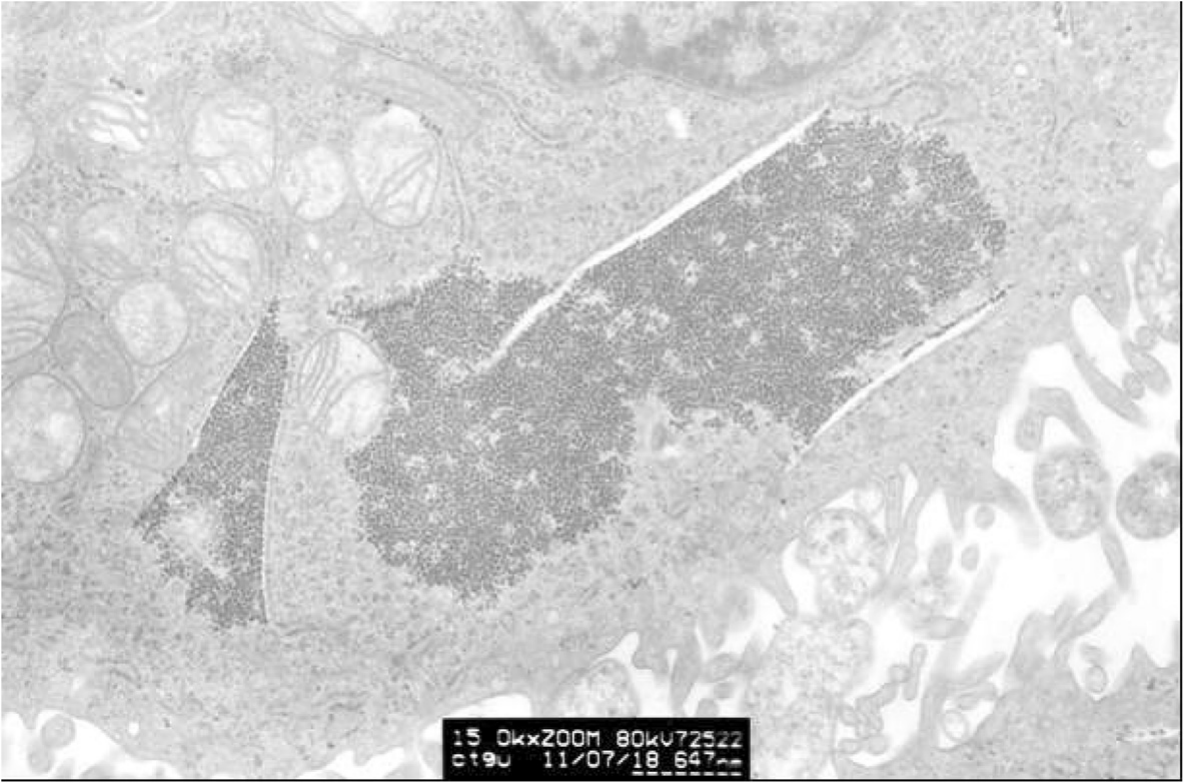
The graph of partial enlargement of the HeLa cell electron micrograph in Fig. 5. Two of the inclusion body structures are highly magnified. At higher magnification, this inclusion body can be seen as a dense aggregation of plenty of tiny granules with uniform diameter

**Fig. 7:**
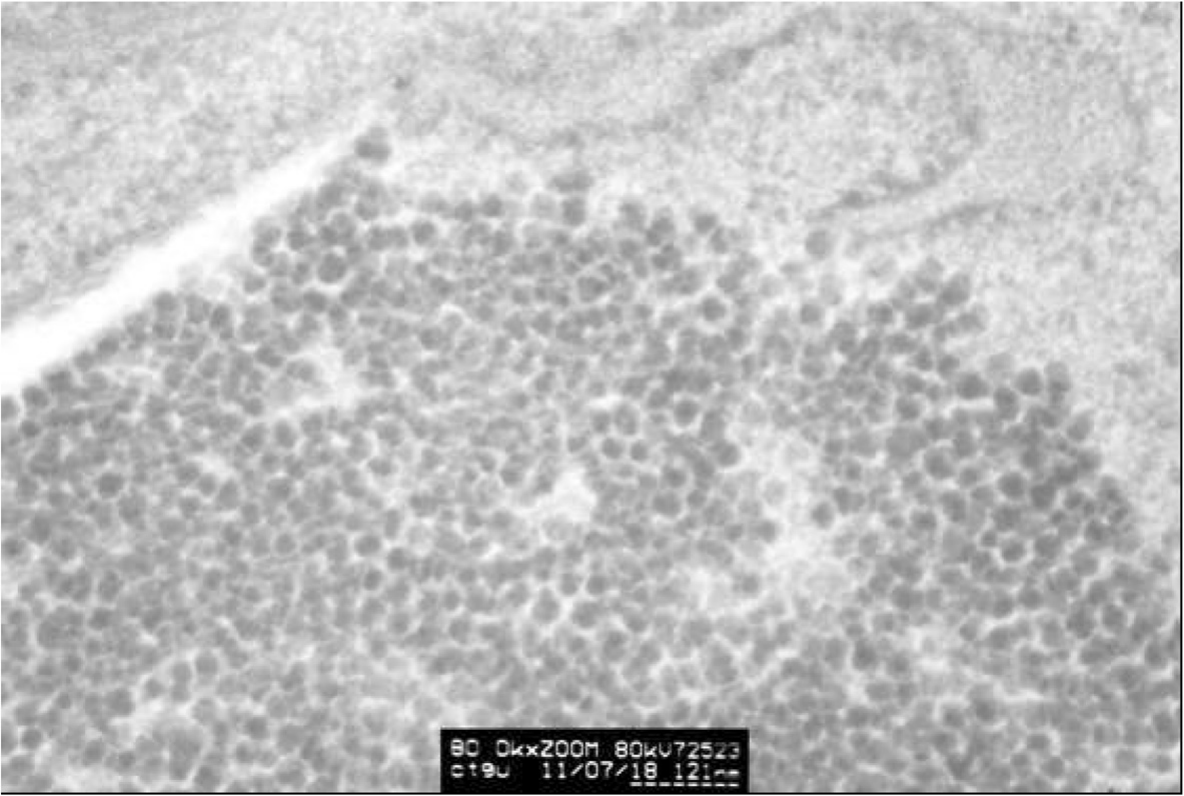
The graph of partial enlargement of the HeLa cell electron micrograph in Fig. 6. Observed at high magnification, these inclusion bodies can be seen as dense aggregations of plenty of round granules with uniform diameter of about 30 nm

Clumped inclusion bodies have been observed in cytoplasm analogue of partial HeLa cells under electron microscope at lower power. These clumped inclusion bodies with different sizes and shapes are not in other cells. The contents of assemble bodies in cells also vary from each other. Some cells have a large number of clumped cytorryctes while some have barely any clumped inclusion bodies. The clustered content is not obviously associated with any cell activity, that is, there is no evident relationship between the clumped content with the growth rate of the cells, whether the cell is in active growth or in apoptosis. At high magnification, it can be seen that these clumpings are not wrapped up by surrounding membranes but rather there are clear dividing lines between the clumped inclusion bodies and other components in the cytoplasm. These clumped inclusion bodies are densely assembled by plenty of round granules that are tiny, with diameter of about 25–30 nm and uniform size, seen scattered in the cytoplasm and nucleus. No inclusion bodies have been observed in the cell nucleus (Figs. 3, 4, 5, 6 and 7).

**Fig. 8:**
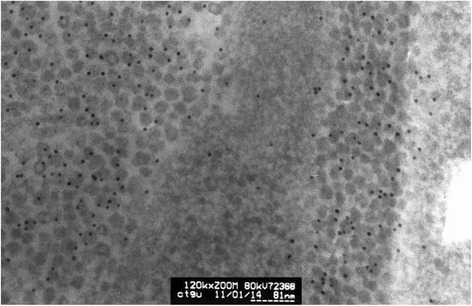
HeLa cell electron microscopy immunocytochemistry stain graph. HeLa cells were labeled using colloidal gold after HPV L1 specific antibody immunocytochemistry reaction. It can be seen that there are dense gold particles in the compact zone of granules which form the inclusion bodies while there are barely any gold particles in the surrounding cytoplasm. This shows that the granules composing the inclusion bodies are HPV 18 L1 or the fusion protein containing HPV18 L1

The HeLa cell ultrathin section was reacted with mice anti-HPV L1 multivalence antibody and then combined with colloidal gold labeled goat anti-mice second antibody. The resulting sample after colloidal gold immunocytochemistry reaction shows the existence of dense gold particles in inclusion bodies zone in the HeLa cell. Large numbers of inclusion body granules appear labeled by colloidal gold particles. However, almost no gold particles are observed in the zone without inclusion body granules. The difference is evident. The scattered granules can be labeled by the colloidal gold. This reaction demonstrates that these granules can sign the specificity of HPV L1 antibody with colloidal gold, which implies that these granules may be HPV L1 protein or fusion protein containing L1 (Fig. 8).

### HPV L1 protein expression assay by Western blotting

**Fig. 9:**
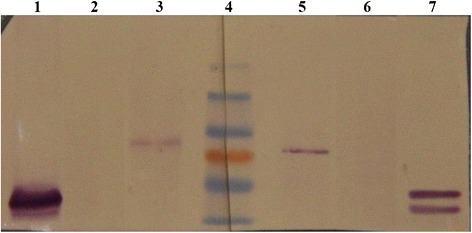
HPV L1 protein expression detection in HeLa cells by Western blotting. Lane 4 is Maker, the molecular weight of which are respectively 55, 72(red), 95, 130 kDa. Lanes 1 to 3 are the results of rabbit anti-HPV L1 broad spectrum polyclonal antibody reaction. Lanes 5 to 7 show the results of mice anti-HPV L1 multivalence monoclonal antibody reaction. Lane 1 and 7 are HPV18 L1 recombinant protein. Lane 2 and 6 are HaCat cell lysates. Lane 3 and 5 are HeLa cell lysates

The results of this assay showed that there were puce colored positive stripes appearing near 55 kDa when recombinant HPV18 L1 protein was reacted with rabbit broad spectrum anti-HPV L1 polyclonal antibody and mice anti-HPV L1 multivalence monoclonal antibody, respectively. However, for the HeLa cell lysates, the positive stripes appeared at 80–85 kDa when reacted with these two types of antibodies. There was no stripe in immortalized keratinocyte – the HaCat cell which is the negative control. This demonstrates that there is HPV L1 protein expression in HeLa cells, despite the possibility that it may not be pure L1 protein but a kind of fusion protein containing HPV L1 (Fig. 9).

## Discussion

It is generally acknowledged that HPV L1 protein expression is associated with the maturation process from basal cells to superficial epithelial cells. Complete life cycle of HPV with whole virus genome only appears in the differentiating keratinocyte [9,11–13,21]. Thus, HPV L1 can only be expressed in the superface cells of squamous epithelium and can be highly expressed in low-grade cervix epithelium diseases. In high-grade diseases, virus DNA integrates to the host DNA and the upstream regulation sequence of HPV L1 gene is lost because of the rearrangement of virus genome, which suppresses the L1 gene transcription. This results in halted production of L1 mRNA, which means that the L1 protein cannot be expressed [11–13,15,16]. Moreover, it is widely considered that L1 protein is dependent on cell differentiation, and even though the tumor cells have intact HPV episomes, the L1 gene expression program is unable to start because of the dedifferentiation of the tumor cells, which results in no expression of L1 protein. However, it is not clear as to whether this viewpoint is applicable to adenocarcinomas infected by viruses including HPV18, or 45.

Previous studies have indicated that is the possibility of L1 expression in the tissue cells of tumors caused by HPV is not only connected to the differentiation state of the tumor tissue but also related to the host genome position integrated by HPV virus genome [22–24]. Although HPV18 virus genome is likely to lose partial genome when it is integrating, several studies have revealed that the loss of L1 genome is minimal, hence keeping it complete [17,18,25]. It is well-known that two integrated HPV18 virus genomes exist in the HeLa cell, and they are 7.8 kb and 6.9 kb respectively [18]. Because there is a 3’ splice site at the beginning of the remaining HPV L1 gene and a late polyA signal behind the gene [26,27], when the inserted site of the HPV18 genome is right in the transcription sequence of host genome and in a suitable internal environment of cells, the HPV18 L1 is likely to be transcribed and spliced appropriately to express L1 protein. Some researchers have discovered the existence of HPV transcript derived from host chromosome and also transcript of L1 when they analyzed the HPV genetic transcripts in HPV integrated tumor tissue [22,23]. The research reported in this paper utilized RT-PCR to detect the existence of HPV L1 transcript in HeLa cells. Our results demonstrated that the L1 genetic transcription of HPV genome integrated in HeLa cells can be detected.

When HeLa cells were observed under electron microscope, some inclusion bodies were found with different numbers, sizes and shapes in the cells cytoplasm. This research elucidated the nature of these materials. ELISA, immunocytochemistry, electron microscope immunocytochemistry and Western blotting were used to demonstrate that there is HPV L1 expression in HeLa cells and that the HPV L1 exists as granules with same size which form the inclusion bodies in the cytoplasm of HeLa cells. These inclusion bodies are composed of large number of tiny granules with similar shapes, uniform diameter about 25–35 nm and bigger electron density. These granules can bind to anti-HPV L1 antibody and be labeled by secondary antibody with colloidal gold. Although they are composed by HPV L1, it is difficult to figure out morphologically what material constitutes these granules. Previous researches have shown that formed L1 protein monomers can assemble to pentamer capsomeres automatically and further form into whole viral shells or full-size virus-like particles (VLP) about 55 nm in diameter, all comprising of 72 pentamer capsomeres [28]. When 10 amino acid residues are truncated in the beginning of L1 N-terminal, the pentamers may only assemble to small VLP about 30 nm in diameter which is composed of 12 pentamers [28,29]. However, when there is much loss of amino acid residue in the L1 C-terminal sequence, the pentamer capsomeres fail to assemble into viral shell or VLP [28,29]. In this research, sophisticated structure of the granules labeled by colloidal gold under the electron microscope is barely visible owning to the quality of the microscope filmmaking, but it can be determined that its diameter is about 25–35 nm. In this research, Western blotting detection shows that the positive reaction site of L1 expressed in HeLa cells appears between 80–85 kDa, which is remarkably higher than the normal site of L1 at 56 kDa. This indicates that the expressed L1 protein in HeLa cells is more likely to be a kind of fusion protein. Further research is needed to determine the exact type of protein that is fused with HPV L1.

Clinical research has shown that the HPV L1 protein expresses in the tumor cells. Researchers such as Lee [30] performed HPV L1 detection for 102 cervix cancer patients and found that HPV L1 protein can be expressed in cervix cancer with 40 % expression rate as well as low grade squamous intraepithelial lesions and high grade squamous intraepithelial lesions (expression rate is about 75.8 and 40 %, respectively). Our research team has conducted investigation to determine HPV L1 expression in cervix tissue. We have found that there is HPV L1 expression in tumor tissue, and HPV L1 expression in some tissues is related to the differentiation of the tumor tissue [24]. These researches demonstrate that HPV L1 expression in tumor tissue or tumor cells is not a rare event.

The results of this present research may be entirely different from mainstream view formed in this research field, but based on research studies and clinical reports, we assume that HPV L1 can be expressed in certain cervix cancer tissues or cells. This expression is highly related to the physical status of virus genome in the tumor tissue or cells (integrated or episomal forms), the differentiation of tumor tissue (high differentiation or low differentiation) and the nature of host genome sequence inserted into by virus genome (can encode or not). We also observed that granules exist not in all HeLa cells. Some cells have lots of granules while some cells have few, hence, there is no definite relationship between the amount of granules and the state of cells (active or in apoptosis). The precise mechanism of L1 expression in HeLa cells is the subject of further research in the future.

The research presented in this paper has two aspects of significance. First, because HPV L1 protein expressed in tumor cells is highly immunogenic, the L1 protein can be used as a target protein for tumor biotherapy. When the present HPV L1 VLP vaccine is used as prophylactic vaccine, it can play a crucial role as the therapeutic vaccine to tumors with L1 protein expression of corresponding type as well. Next, if there is L1 expressed in tumor cells, L1 can work as tumor antigen and it is beneficial for body's immune system to recognize and remove these tumors. It is also possible that cancer patients with L1 expression may be able get better prognosis than those with no L1 expression [31].

## Methods

### Reagents

HeLa cells and immortalization keratinocytes HaCat cells (negative control cell line) were purchased from China Center for Type Culture Collection (Wuhan University). DMEM cell culture containing 10 % fetal calf serum was cultivated using routine method. TRNzol total RNA extraction kit and cDNA first chain synthesis kit were bought from Tiangen Biotechnology (Beijing) Company Limited. The primers of MY09/11 (CGTCCMARRGGAWACTGATC, GCMCAGGGWCATAAYAATGG) and the primers of human GAPDH (positive sense: AGAAGGCTGGGGCTCATTTG, antisense: AGGGGCCATCCACAGTCTTC) were synthesized by Sangon Biotechnology Company Limited (Shanghai). The mice anti-HPV1, 6, 11, 16, 18, 31 L1 multivalence monoclonal antibodies were purchased from Millipore Corporation (MAB837). Rabbit broad spectrum anti-HPV L1 multiclone purified antibody [32] was produced by HangZhou HuaAn Biotechnology Company Limited. Ф10nm colloidal gold labeled goat anti-mice IgG was the product of Sigma Corporation (G7652). Biotin-Antibiotin-Horseradish peroxidase compounds and biotinylation goat anti-mice IgG were bought from Wuhan Guge Biotechnology Company Limited. Mice antibody immunocytochemistry kit was the product of Neo Bioscience Technology Company Limited. Protein Marker was produced by Fermentas (SM0672). Recombinant protein HPV18 L1 expressed by Sf9 insect cells baculovirus expression system was a gift from professor Xu Xue-mei who is working for the Institute of Basic Medical Sciences, Chinese Academy of Medical Sciences [33].

### HeLa cells and HaCat cells total protein and mRNA extraction

Total protein was extracted from HeLa cells and HaCat cells by routine method of mono-decontaminate lysate. Extracted protein was sub-packaged into a 0.5 ml centrifuge tube and was preserved at − 20 °C. The total mRNA was extracted by TRNzol total RNA extraction kit procedure while the cells were cultivated and preserved at − 70 °C.

### HPV L1 protein expression assay by ELISA

Appropriate protein sample was transferred into 96 wells ELISA plate, and was diluted with 0.05 M carbonate buffer solution up to 15–20 μg/ml, then it was coated to stay overnight at 4 °C. On the second day, the plate was washed with phosphate buffer solution (PBS), and then blocked by 3 % Bovine Serum Albumin (BSA). After blocking solution was removed, mice HPV L1 antibody was diluted by 1:5000 and added to the wells. Next, the plate was washed with PBS, which was followed by addition of 1:1000 diluted goat anti-mice IgG antibody labeled by horse radish peroxidase and incubating at 37 °C for 1 h. After being washed with PBS and colored by TMB chromogenic solution, the reaction was terminated by adding 2 M H_2_SO_4_. Then, the optical density values were read by 450 nm microplate reader and the experiments were repeated in 3 replicates to get the average value. The obtained optical density (OD) values were calculated using the formula (sample OD value-blank) divided by (negative-blank), so the calculated value of ≥2.1 means that it is a positive reaction. Software SPSS13 was used to get the average values $\left(\overline{x}\pm \mathrm{S}\right)$ and to carry out single factor variance analysis in order to calculate the P value.

### Reverse transcription polymerase chain reaction (RT-PCR) assay

The first chain cDNA was synthetized by the cDNA first chain synthesis kit using HeLa cells total mRNA. PCR amplification reaction was done using 10 % of the first chain cDNA reaction liquid. Amplification primer used was the HPV L1 universal primer MY09/11 and the products were about 450 bp. PCR products were processed by agarose gel electrophoresis and the pictures were taken by gel imaging system. GAPDH was used as a internal control. The products were 258 bp.

### HPV L1 protein expression assay by light microscope immunocytochemistry

Cell slides with fixed HeLa cells and HaCat cells were put into a glass plate pool. It was washed with PBS which was followed by addition of 3 % H_2_O_2_ to remove endogenous peroxidase. After washing with PBS again, anti-HPV 1, 6, 11, 16, 18, 31 L1 monoclonal antibody (concentration 1:1500) was added to the cells and incubated for 1 h. After another wash with PBS, biotinylation goat anti-mice IgG second antibody is added to the system which was following by a wash with PBS and addition of Biotin-Antibiotin-Horseradish peroxidase compounds. The system was then washed with PBS, stained by DAB chromogenic solution, then washed with PBS and counterstained with hematoxylin. Finally, the cell sample was rinsed with water, then dehydrated, cleared, gum sealed and examined by light microscopy.

### HPV L1 protein expression assay by electron microscope immunocytochemistry

Logarithmic phase growing HeLa cells were collected by centrifugation after they were digested by 0.5 % trypsin. The sedimentation was fixed by 1.0 % glutaraldehyde and was made into ultrathin sections under transmission electron microscope. The ultrathin sections were put in the nickel network and etched with 10 % H_2_O_2_ for 10 min. After washed with PBS, 1:500 mice anti-HPV1, 6, 11, 16, 18, 31 L1 monoclonal antibody and 1:200 Ф10nm gold colloid labeled goat anti-mice IgG were added to the section. When the reaction reached completion, it was counterstained by uranium and lead, followed by the examination of the sample under Hitachi HT-7500 transmission electron microscope.

### HPV L1 protein expression assay by Western blotting

Ready-processed protein samples and recombinant HPV18 L1 protein were added into SDS-PAGE gel sample holes for electrophoresis, and then the gel was protein transferred. After protein transfer, the NC membrane was treated with 3 % BSA blocking buffer solution, then detected by mice anti-HPV1, 6, 11, 16, 18, 31 L1 monoclonal antibody. The mice monoclonal antibody was diluted by TBS solution containing 5 % calf serum to 1:10000 and then was oscillated with NC membrane for 2 h. After being washed with TBS, the hatched membrane was oscillated with HRP labeled goat anti-mice IgG diluted to 1:4000 by TBS (which contains 5 % calf serum). The membrane washed by TBS was then stained by DAB stain solution. Lastly, double distilled water was used to terminate the reaction and the membrane was taken out to dry and take pictures. In the meantime, rabbit anti-HPV L1 antibody was used to do the same detection with the same procedure as mice monoclonal antibody.

## Abbreviations

HPV: Human papillomavirus
L1: Major capsid protein
RT-PCR: Reverse transcription polymerase chain reaction
GAPDH: Reduced glyceraldehyde-phosphate dehydrogenase
ELISA: Enzyme-linked immuno sorbent assay
VLP: Virus-like particles
